# A gene-specific non-enhancer sequence is critical for expression from the promoter of the small heat shock protein gene *αB-crystallin*

**DOI:** 10.1186/1479-7364-8-5

**Published:** 2014-03-03

**Authors:** Zhe Jing, Rajendra K Gangalum, Dennis C Mock, Suraj P Bhat

**Affiliations:** 1 Jules Stein Eye Institute, University of California, Los Angeles, CA 90095, USA; 2 Geffen School of Medicine at UCLA, Brain Research Institute and Molecular Biology Institute, University of California, 100-Stein Plaza, Rm BH 623, Los Angeles, CA 90095-700019, USA

**Keywords:** Gene-specific promoter sequence, Gene expression, *αB-crystallin*, Human retinal pigment epithelial cells, Transgenic mice

## Abstract

**Background:**

Deciphering of the information content of eukaryotic promoters has remained confined to universal landmarks and conserved sequence elements such as enhancers and transcription factor binding motifs, which are considered sufficient for gene activation and regulation. Gene-specific sequences, interspersed between the canonical transacting factor binding sites or adjoining them within a promoter, are generally taken to be devoid of any regulatory information and have therefore been largely ignored. An unanswered question therefore is, do gene-specific sequences within a eukaryotic promoter have a role in gene activation? Here, we present an exhaustive experimental analysis of a gene-specific sequence adjoining the heat shock element (HSE) in the proximal promoter of the small heat shock protein gene, *αB-crystallin* (*cryab*). These sequences are highly conserved between the rodents and the humans.

**Results:**

Using human retinal pigment epithelial cells in culture as the host, we have identified a 10-bp gene-specific promoter sequence (GPS), which, unlike an enhancer, controls expression from the promoter of this gene, only when in appropriate position and orientation. Notably, the data suggests that GPS in comparison with the HSE works in a context-independent fashion. Additionally, when moved upstream, about a nucleosome length of DNA (−154 bp) from the transcription start site (TSS), the activity of the promoter is markedly inhibited, suggesting its involvement in local promoter access. Importantly, we demonstrate that deletion of the GPS results in complete loss of *cryab* promoter activity in transgenic mice.

**Conclusions:**

These data suggest that gene-specific sequences such as the GPS, identified here, may have critical roles in regulating gene-specific activity from eukaryotic promoters.

## Background

A eukaryotic promoter is heterogeneous in structure. It contains multiple transacting factor binding sites that are shared amongst multiple genes, yet it contains specific information for how and when a gene should be active. Investigations on eukaryotic promoters have sought a common mechanistic thread in cis-regulatory modules of enhancer sequences and transcription factor binding sites (both distant as well as proximal) for an understanding of the control of gene expression [1-3]. There is, however, a finite number of transcription factors that are shared among a large number of promoters [4] (at least 70,000 promoters and 1,800 transcription factors) [5]; thus, combinatorial schemes have been invoked to explain specific gene activation via a ‘regulatory grammar’ that remains to be deciphered [6-9]. Thus, there is no known concrete mechanistic detail that explains the control of specific gene activity [10].

Our understanding of the regulatory information in the eukaryotic promoters has largely come from functional understanding of the shared presence of universal or conserved sequence elements in different genes [1-3,11,12] and has established a major role for transcription factors (transacting factors, coactivators, and basal factors) and their binding sequences in the regulation of gene activity [4,6,13,14]. The import of gene-specific sequences, if any, in the regulation has thus remained uninvestigated. While the commonality of the sequence elements in the promoters of various genes has contributed to the identification and validation of shared sequence motifs experimentally as well as computationally, these approaches, however, cannot be meaningfully applied for the elucidation of the role of gene-specific promoter sequences. At this time, the role of gene-specific sequences can only be determined experimentally, on a gene to gene basis. In this investigation, we have examined one such gene-specific sequence adjoining the heat shock element (HSE) in the proximal promoter of the small heat shock protein gene *αB-crystallin* (*cryab*) and found it to be essential for expression both in cultured cells as well as in transgenic mice.

*Cryab* is the archetypical, conserved, small heat shock protein gene expressed ubiquitously in multiple tissues in vertebrates in a developmentally dictated fashion. Its expression attends a host of pathologies ranging from cardiomyopathies and cataracts to oncogenesis and neurodegenerations such as Alzheimer's disease, multiple sclerosis, and age-related macular degeneration [15,16]. In specific cell types, in culture, it is also expressed in response to heat and osmotic stress [17-19].

**Figure 1:**
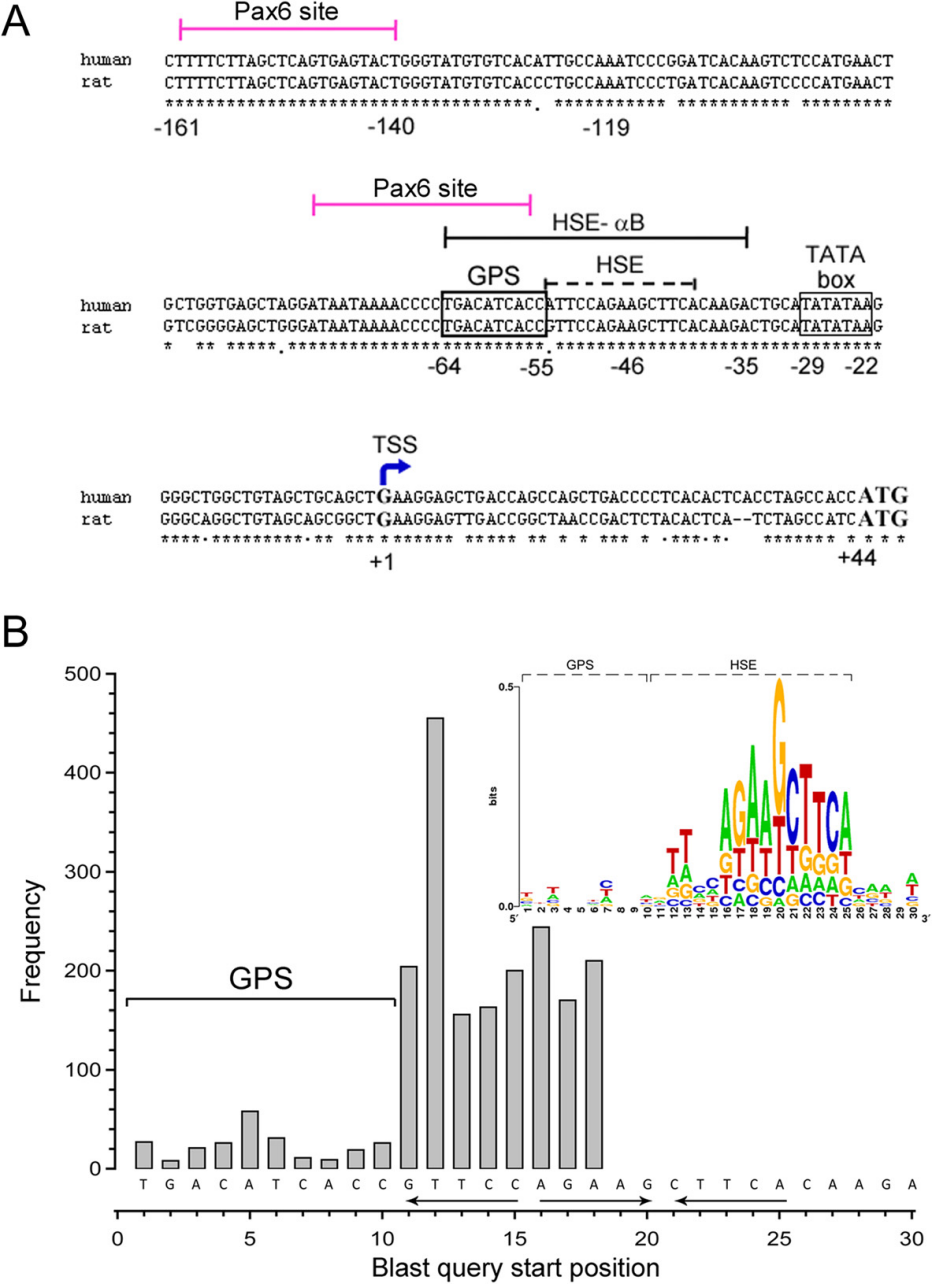
**The location of HSE-αB within the proximal promoter (−162/+44) of the *cryab *gene. (A)** Human and rat sequences in the proximal promoter of the *cryab* gene are highly conserved. The numbering shown *above* is for the rat sequence [20], TSS, transcription start site (*thick blue arrow*). The promoter element HSE-αB (*black line*), a 30-nucleotide promoter sequence, was used for earlier gel-shift studies [24,25]. The TATA box (*thin line*) and the GPS (determined in this investigation) are boxed (*thick line*). The positions of two Pax6 sites [22] (*pink*) are shown. **(B)** A graphic representation of the composition of HSE-αB (−64/−35). This plot was generated from the results of the BLASTN homology search of the human genomic database using HSE-αB (*x-axis*), as the query sequence. Various parts of this sequence pick up homologous DNA sequences starting with various start positions. We calculated the frequency of each start position. The *y*-axis shows the frequency of start positions with homology in the 30-bp query sequence. Note that about 90% of the homology searches start from position 11 of this sequence. Position 11 is the first base of the 5*'*-NGAAN-3′ pentamer, the first of the three inverted sequence motifs (*arrows*) that make the HSE. There were no significant searches that start after position 18. The inset contains a plot of relative information content per base (bits) at each of the 30 positions in the HSE-αB, derived from top 100 BLASTN hits using http://weblogo.berkeley.edu. Based on this analysis, we designate the first 10 bp as gene-specific promoter sequence (GPS), while sequences from positions 11 to 25 constitute the canonical HSE (15 bp) with high-frequency representation in the genome. This analysis is based on 3,336 Blast hits using a maximum number of sequences = 20,000 in the NCBI BLASTN program.

A number of cis-regulatory elements including various enhancers that regulate the expression of the *cryab* gene in different tissues have been previously identified [20-23]. We have characterized the heat shock promoter of this gene [18,24,25], which is highly conserved between rodents and humans (see Figure 1A). It contains a canonical trimeric HSE at −54/−40, which binds the heat shock transcription factor 4 (HSF4) [25]. The HSE is part of the sequence named HSE-αB, (Figure 1), a 30-bp promoter fragment (−64/−35), which has been used previously for HSF4 binding assays in gel-shift experiments [24,25]. The canonical HSE (15 bp) in the HSE-αB is flanked by gene-specific 10-bp on the 5′ end and 5 bp on the 3′ end (Figure 1). Figure 1B graphically defines the 10-bp gene-specific sequence in comparison with a universal sequence motif such as a transcription factor binding sequence like HSE (there are many consensus HSEs in many genes); the 10-bp gene-specific sequence adjoining the HSE on its 5′ end is unique and is only present in the *cryab* gene. In this investigation, we have examined the role of the gene-specific sequences surrounding the canonical HSE (that makes the HSE-αB sequence, −64/−35, Figure 1), in regulating the *cryab* promoter activity in cultured human adult retinal pigment epithelial cells (ARPE-19) and in transgenic mice.

## Results

### A 10-bp sequence in the *cryab* promoter is required for expression in cultured ARPE cells

**Figure 2:**
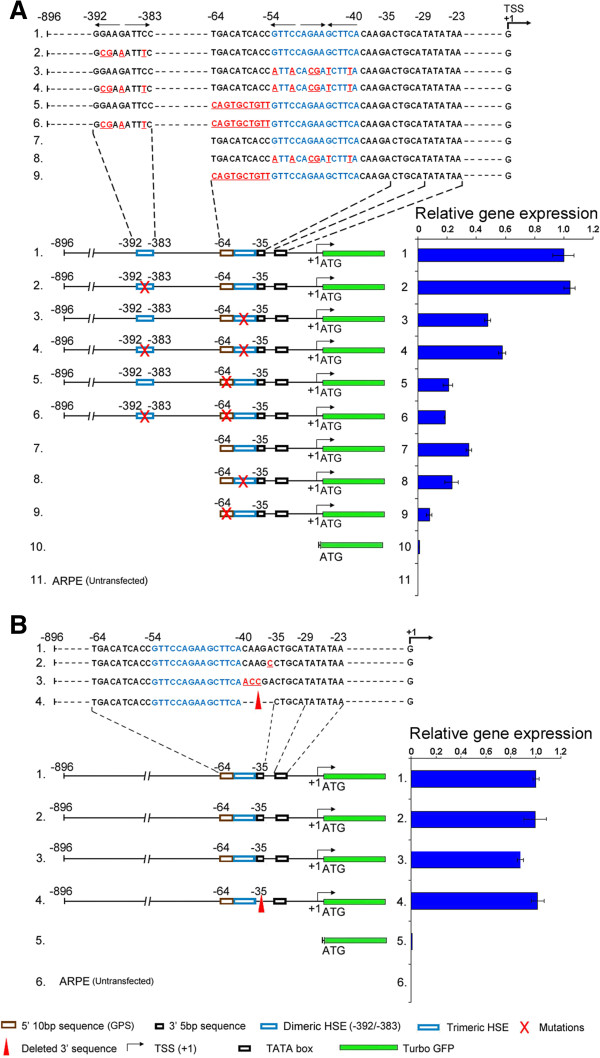
**The 5′ 10-bp sequence adjoining the HSE controls the activity of the *cryab *promoter. (A)***Cryab* promoter sequences (−896/+1), showing the dimeric HSE (−392/−383) and sequences from −64 to −23 that includes the trimeric HSE (*numbers 1–6*). The *thin arrows on top* of the trimeric HSE (*blue*) indicate the 5*'*-NGAAN-3*'* inverted orientation of the HSE motifs. Mutations are indicated in *red* and *underlined* (*numbers 2–6, 8, 9*). The complete promoter is −896 to +44, and the truncated promoter includes sequences −64 to +44. tGFP (*green*) starts with ATG. TSS is shown with an *arrow* at +1. The dimeric HSE (−392/−383) and sequences at −64/−35 (HSE-αB) and −29/−23 (TATA box) are schematically represented with *open boxes* in the promoter/reporter constructs. The corresponding activity of each construct (relative tGFP expression as assessed by RT-qPCR) is shown on the *right* (means ± standard deviation). Note inhibition of tGFP expression (*numbers 5, 6, 9*) by mutations in the 10-bp sequence (−64/−55) adjoining the HSE. The numbers (*1 − 9*) in the *top panel* with relevant sequences correspond to numbers for promoter/reporter constructions in the *lower panel*. Note that deletion of the dimeric HSE at −392/−383 does not affect the reporter expression in these cells (*1 and 2*). **(B)** Sequences on the 3*'* end of the trimeric HSE do not impact the expression significantly. Relevant sequences are shown on *top*. Mutations are indicated in *red* and are *underlined*. Schematics of various constructions and the activity of each promoter/reporter construct, as assessed by RT-qPCR (means ± standard deviation), is shown as in **A**. The key to various notations in **A** and **B** is given on the *bottom*.

ARPE-19 is an established non-transformed epithelial cell line, which naturally expresses αB-crystallin [26]. As expected [24,25], in transient transfections of ARPE-19, promoter-reporter constructs containing mutations in the HSE (−54/−40) reduced expression from the *cryab* promoter appreciably. Both the complete (long) promoter and the truncated promoter were used. We mutated the sequences surrounding the HSE and followed the expression of tGFP. Mutating or deleting the 5-bp sequence from the 3′ end of the HSE does not impact expression appreciably (Figure 2B). Interestingly, however, mutating the 5′ gene-specific 10-bp sequence (−64/−55) adjoining the HSE resulted in a more pronounced inhibition than mutating the HSE (Figure 2A). In the typical experiment shown in Figure 2A, mutations in trimeric HSE reduce the expression by 40%–50% (Figure 2A, numbers 1–4), while mutations in the 10-bp sequence adjoining the HSE inhibit expression by 70%–80% (Figure 2A, numbers 1, 5, and 6; note that mutating the dimeric HSE at −392/−383 did not alter these results). This pattern of the inhibition of the promoter activity, obtained with mutated 10-bp sequence adjoining the HSE was consistent both when using the complete (−896/+44; Figure 2A, constructs 1–6) as well as the truncated promoter (−64/+44; Figure 2A, constructs 7–9). This is clearly an antithesis of what would be expected because the 10-bp sequence is a gene-specific sequence adjoining the canonical HSE. The canonical HSE binds HSF4 and is present in many heat shock promoters, thus considered to contain regulatory ‘information’. The gene-specific sequence, on the other hand, is only present in the *αB-crystallin* gene and is therefore perceived to be devoid of any regulatory information.

Much against this perception, however, the 10-bp sequence adjoining the HSE seems to work in a context-independent fashion. This is revealed by the observation that the same level of inhibition is obtained when this sequence is mutated within a complete promoter (Figure 2A, numbers 1–6) as well as when it is part of the truncated version of the promoter (Figure 2A, numbers 7–9). In comparison, when HSE (−54/−40) in the truncated promoter is mutated, the inhibition of the expression is not as pronounced as when the whole promoter is used (Figure 2A, compare numbers 1 and 3 and numbers 7 and 8) reiterating the known context-dependent [27] functioning of individual promoter motifs or a transcription factor binding site, in this case, the HSE. We conclude that the 5′ 10-bp sequence (−64/−55) adjoining the HSE contains information that is required for expression from the *cryab* promoter in human ARPE cells.

### The 10-bp sequence functions in a position and orientation-specific fashion

**Figure 3:**
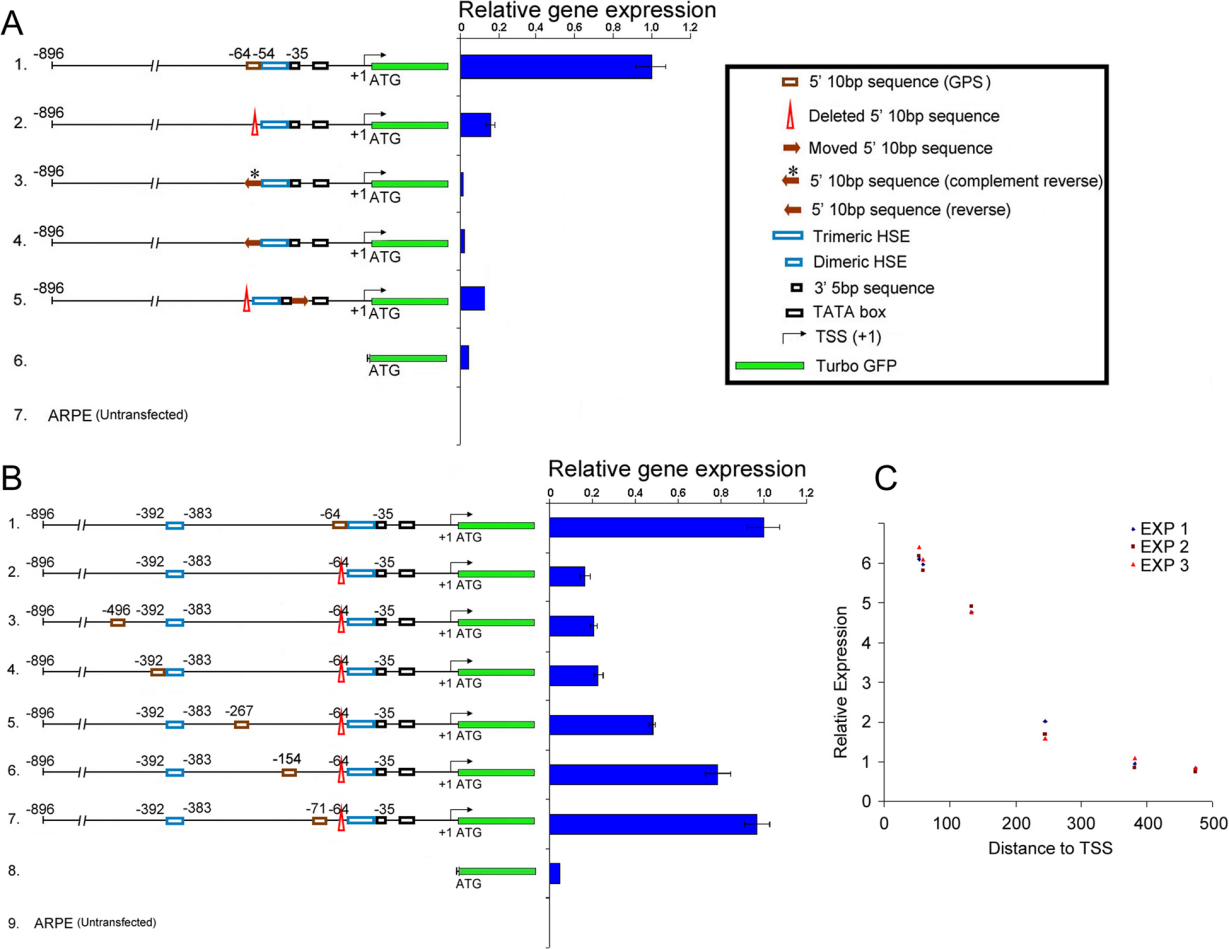
**The 10-bp sequence functions optimally only in appropriate position and orientation.** Schematic representations of various constructions and corresponding activity profiles (RT-qPCR) are shown in **(A)** and **(B)**. **(A)** Complete (−896/+44) *cryab* promoter/tGFP constructs were used. The values shown are the means ± standard deviation. Note that all manipulations of the 10-bp sequence (−64/−55) have a drastic inhibitory effect on tGFP expression. **(B)** Functional consequences (RT-qPCR) of moving GPS upstream to different distances from the TSS. **(C)** A plot of the expression of tGFP as a function of the distance of GPS from TSS. Data from three separate experiments (EXP1-3) is plotted. Note that the response is biphasic, and moving GPS to 5*'* upstream beyond −154 has an inhibitory effect on tGFP expression. The key to various notations is given in the *top right box*.

We further explored the status of the 10-bp sequence (−64/−55), identified above, as an independent promoter element. We deleted it (Figure 3A, number 2), reversed its orientation without changing its position (Figure 3A, numbers 3 and 4), and changed its position without reversing its orientation by placing it on the 3′ end of HSE-αB sequences (Figure 3A, number 5). All of these manipulations inhibit promoter activity (Figure 3A). This data leads to the conclusion that the 10-bp sequence adjoining the HSE on the 5′ end (−64/−55), unlike an enhancer, contains positional information, which is essential for the expression from the *cryab* promoter. We named this sequence as the gene-specific promoter sequence (GPS).

### Impact of GPS on promoter activity when moved to various distances from the transcription start site

The GPS, when moved to the 3′ of the HSE-αB inhibits expression (Figure 3A, number 5). The GPS was also moved to −71, −154, −267, −392, and −496 (with transcription start site (TSS) as +1) which corresponds to 7, 90, 203, 328 and 432 bp upstream from its original position (at −64/−55), respectively (Figure 3B). When moved upstream to −71 or −154 positions, the effect on expression is minimal (Figure 3B, numbers 6 and 7). However, when moved farther than −154 bp from the TSS, there is a precipitous loss of promoter activity (Figure 3B, numbers 3–5).

It is important to note that the movement of the GPS from its original site to a new site (Figure 3B) does not disrupt any essential sequences required for expression. GPS is part of one of the two consensus Pax6 binding sites (−160/−140 and −77/−55) in the *cryab* promoter (Figure 1A) [21-23,28]. Note that moving the GPS from −64 to −71, which disrupts the proximal Pax6 site, hardly impacts the expression (Figure 3B, number 7) as does the placement of the GPS at −154, which disrupts the distal Pax6 site (see Figure 1A), reducing the expression only by about 15% (Figure 3B, number 6) suggesting that neither Pax6 site significantly contributes to the expression from the *cryab* promoter in ARPE cells in culture.

**Figure 4:**
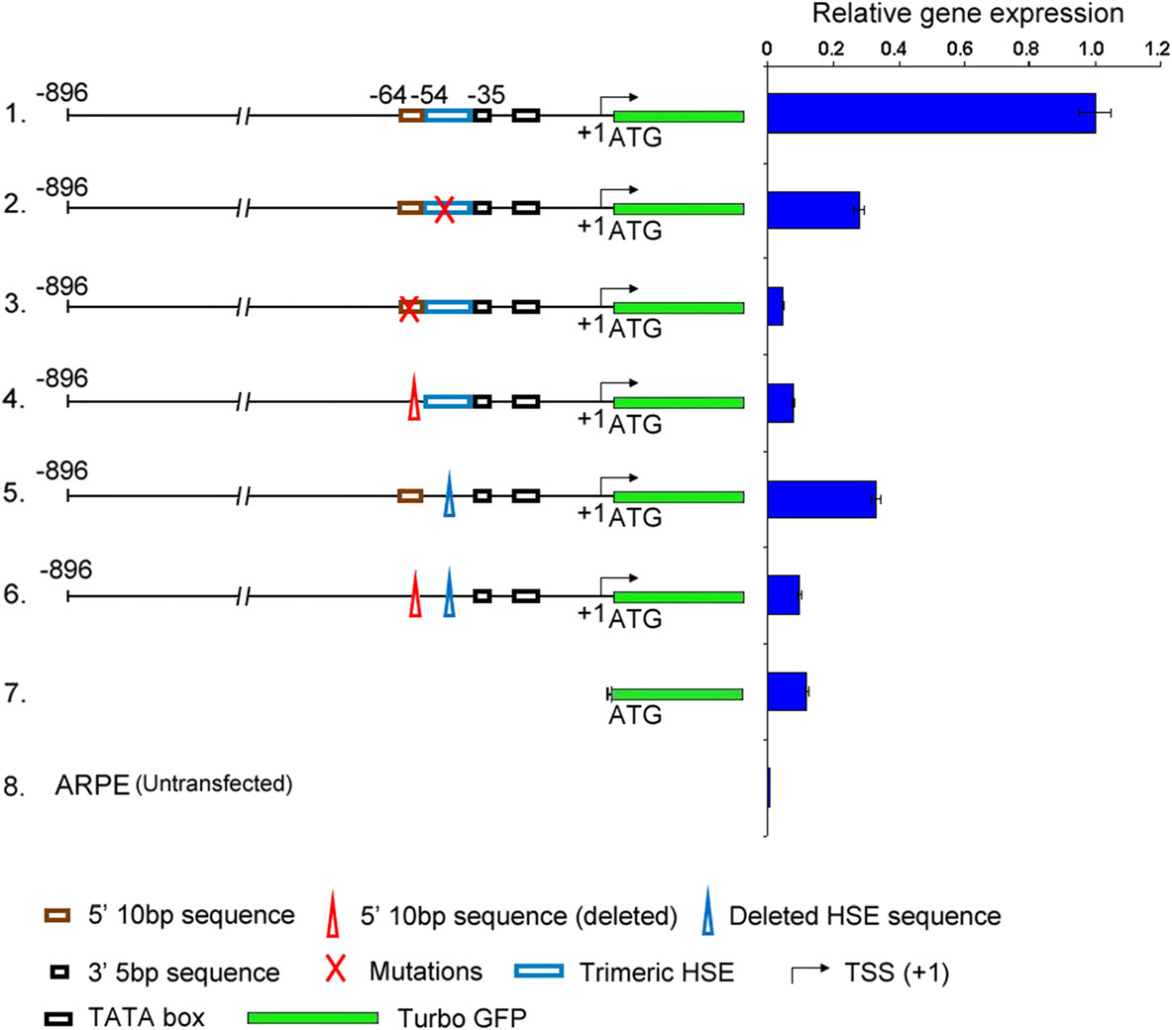
**Comparison of point mutations versus deletions on the tGFP expression driven by *cryab *promoter.** Complete promoter constructions were used as in Figures 2 and 3. Expression of tGFP was assessed by RT-qPCR of total RNA. Note that both the point mutations and deletions (in HSE and GPS) have the same relative effect on the tGFP expression (compare *numbers 2 and 5* and *numbers 3 and 4*). Importantly, alterations in the 10-bp sequence have a more pronounced effect on the tGFP expression than alterations in the HSE (compare *number 2 with 3* and *number 5 with 4*). The construction (*number 6*) contains deletions of both the 10-bp sequence (GPS) as well as the HSE. Interestingly, the effect on activity is similar to that obtained with deletion of the 10-bp sequence (GPS) alone (*number 4*). The constructs numbers 2 and 3 correspond to constructs numbers 3 and 5, respectively, in Figure 2A. The key to the schematics is given at the *bottom*.

In the mouse *cryab* promoter, sequences downstream of −426 have been shown to be enough for expression in transgenic mice [22]; thus, moving the GPS to −496 (Figure 3B, number 3) should have minimal impact on the expression, yet this manipulation also inhibits the expression. Therefore, it is the absence of GPS from its appropriate place rather than its movement to a new place that results in the inhibition of the *cryab* promoter activity. This is further evidenced by the fact that the movement of the GPS to any position (Figure 3B, numbers 3-7), 5′ to its natural position (−64/-55), has a gradual impact on the expression. In comparison, when GPS is moved 20 bp 3′ to its original position (−55), the expression is completely blocked (see Figure 3A, number 5). It is important to note that all the manipulations (point mutations and deletions) show similar results (Figure 4), indicating that structural perturbations, if any, do not affect the promoter activities assayed here (Figures 2 and 3).

### GPS is required for expression in transgenic mice

**Figure 5:**
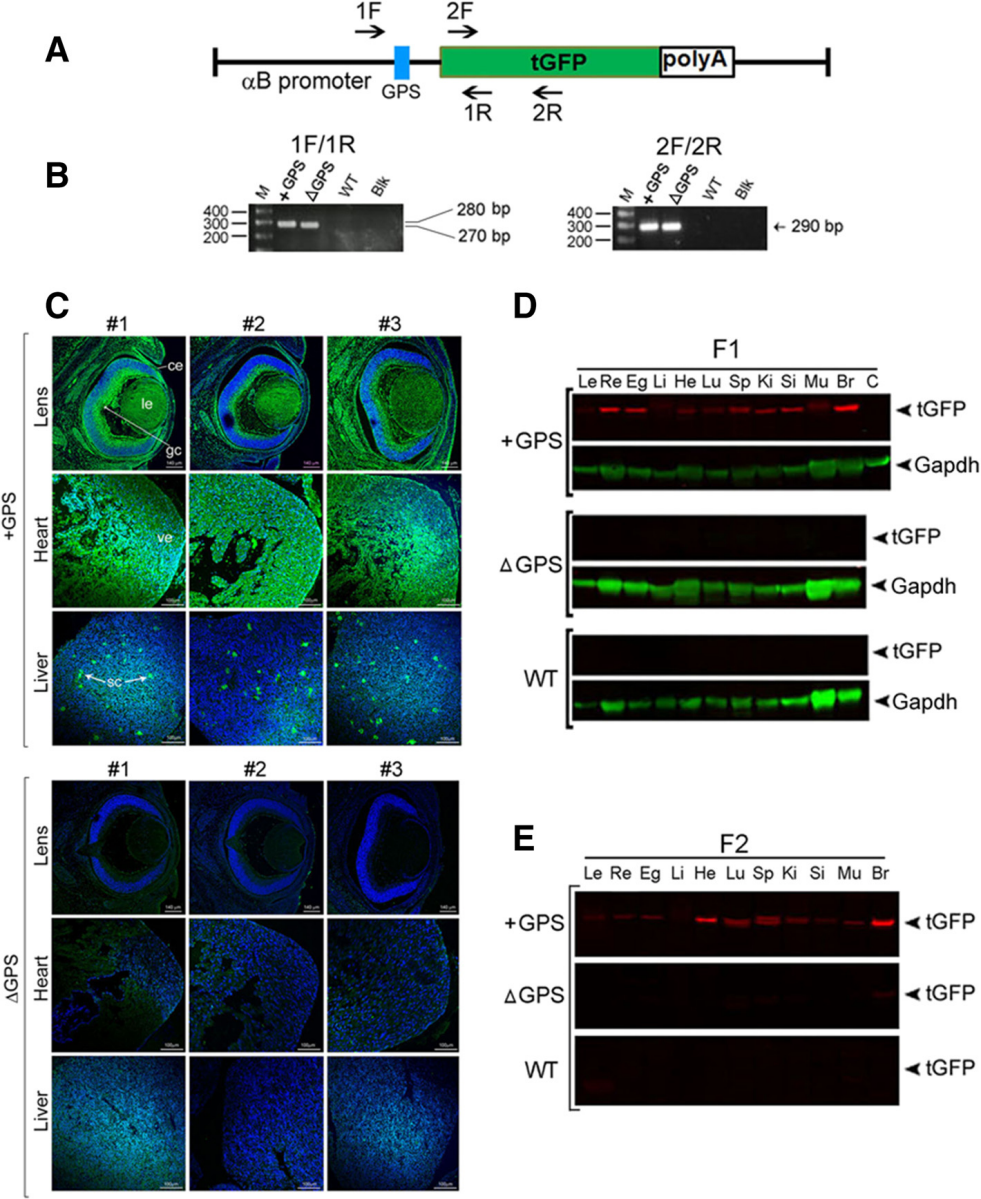
***Cryab *promoter without GPS (ΔGPS) is inactive in transgenic mice. (A)** Schematic of the + GPS *cryab* promoter-tGFP transgene (*GPS*, *blue rectangle*). Primer locations for genotyping are shown. **(B)** Genotyping of transgenic mice, *F* forward primer, *R* reverse primer. Note the lower mobility of the PCR product (270 bp, *left panel*) generated from ΔGPS mice in comparison with the (+)GPS mice (280 bp). The *right panel* (with *2F* and *2R*, internal for tGFP) shows no change in amplicon size (290 bp). *WT* wild-type non-transgenic DNA, *Blk* blank. **(C)** Expression of tGFP (*anti-tGFP immunofluorescence*, *confocal images*) in three transgenic mouse lines (*numbers 1–3*) in + GPS and ΔGPS mice. Middle *z* sections of three tissues (*eye*, *heart*, and *liver*) are shown from + GPS (*top*) and ΔGPS mice (*bottom*). In + GPS transgenic tissues, tGFP immunofluorescence is obvious in both the developing heart (*ve* ventricle) and the eye (ocular lens (*le*), corneal epithelium (*ce*), and ganglion cells (*gc*)). In addition, tGFP expression is seen in the surrounding choroid and mesenchymal cells between the lens and the developing retina. The specific expression of tGFP in hepatic stellate cells (*sc*) is striking (*+GPS*, *liver*). Note the absence of expression (immunofluorescence) in the ΔGPS lines in all tissues (*bottom*). **(D)** Immunoblots of 11 tissue extracts of F1, (+)GPS and ΔGPS (transgenic line 2), and WT. Gapdh *green bands* (*internal control*). *Le* lens, *Re* retina, *Eg* eye globe without the lens and retina, *Li* liver, *He* heart, *Lu* lung, *Sp* spleen, *Ki* kidney, *Si* small intestine, *Mu* muscle, *Br* brain, *C* control ARPE-19 cell extract. **(E)** Immunoblots of tissue extracts as in **(D)** from F2, line 2 transgenic animals. Note that the WT and ΔGPS tissues do not show any reactivity for tGFP.

We next ascertained if GPS controls the expression from the *cryab* promoter in the whole animal. Transgenic mice were produced with complete rat *cryab* promoter-turbo green fluorescent protein (tGFP) constructs (see Figure 5A) with (+GPS) and without GPS (ΔGPS). αB-crystallin is expressed very early in the developing heart and the ocular lens [29,30]. Accordingly, in transgenic mice containing the wild-type promoter (+GPS), the expression of tGFP as detected by immunohistochemistry is seen in the developing eye and the heart. In animals made without the GPS (ΔGPS), no tGFP expression is seen (Figure 5C, bottom panels). In Figure 5C, confocal images of middle *z* sections from three tissues each (eye, heart, and liver) from three transgenic lines (numbers 1–3) have been shown. Interestingly, in the liver, αB is known to be expressed only in stellate cells [31]. This is confirmed here by the specific detection of tGFP in these cells in the livers of transgenic mice containing GPS (+GPS). In transgenic animals produced without the GPS (ΔGPS), the tGFP expression is absent (Figure 5C). The immunohistochemistry data was further corroborated by immunoblotting of 11 different tissues from the F1 (Figure 5D) and F2 generation transgenic mice (Figure 5E). This data establishes that no tGFP transgene expression is detected in transgenic animals generated with recombinant constructs without GPS (ΔGPS) (Figure 5).

**Figure 6:**
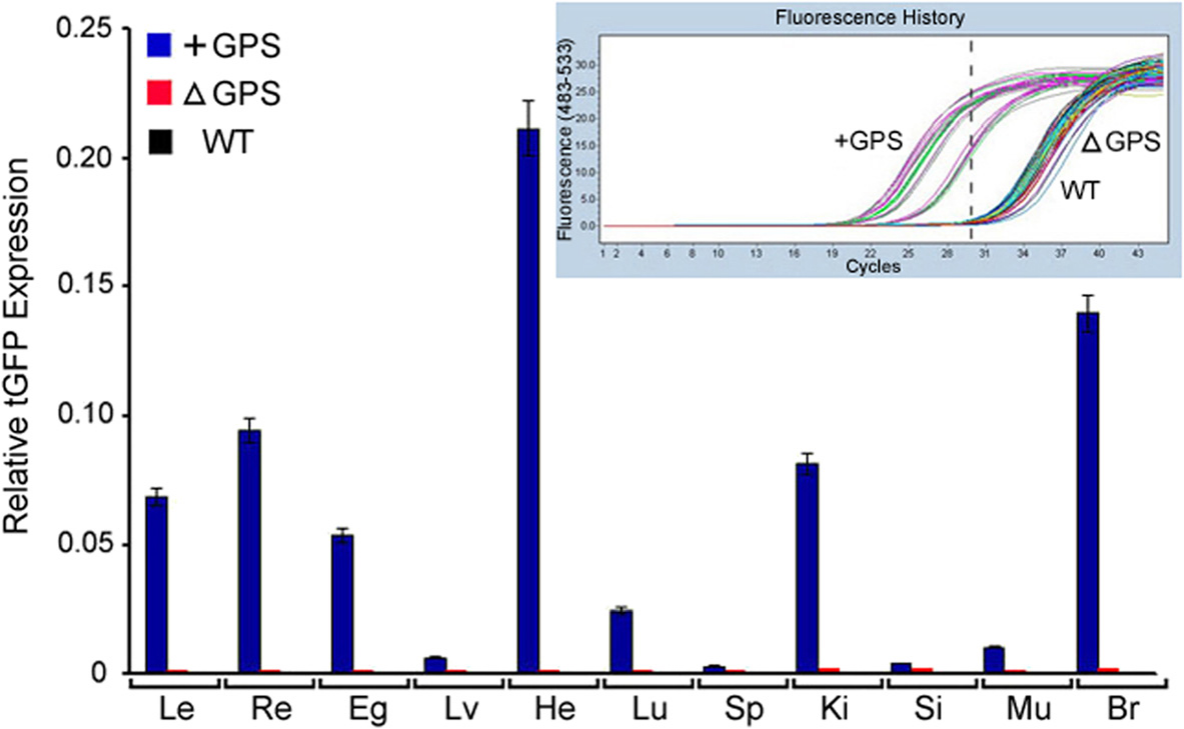
**GPS is essential for the *cryab *promoter activity in transgenic mice.** Relative tGFP expression was assayed in transgenic mice with GPS (*+GPS*), without GPS (*ΔGPS*) and wild type non-transgenic mice (*WT*). RT-qPCR analysis of tGFP expression in 11 mouse tissues is shown. Two transgenic lines and two WTs were analyzed, but no tissues were pooled. No tGFP is seen in ΔGPS and WT animals. *Le* lens, *Re* retina, *Eg* eye globe without the lens and retina, *Li* liver, *He* heart, *Lu* lung, *Sp* spleen, *Ki* kidney, *Si* small intestine, *Mu* muscle, *Br* brain. The *inset* is a screen shot of the raw data from the 480 cycler (Roche).The *dotted vertical line* separates the + GPS samples from the ΔGPS and WT samples.

αB-crystallin is known to be expressed at high levels in the lens, but the tGFP protein levels detected in the transgenic lens are much lower (immunoblots in Figure 5D,E). However, it is the tGFP transcript levels that should be considered more relevant to the expression than the absolute amount of tGFP protein. The detection of tGFP protein levels may be masked by normally high concentrations of crystallin proteins in the lens and/or poor translation of the transgenic mRNA in comparison with endogenous *cryab* mRNA. Importantly, the RT-qPCR detects high levels of tGFP transcripts in the lens, retina, heart, kidney, and the brain in + GPS animals (Figure 6). However, the absolute requirement for the presence of GPS in the *cryab* promoter is established by the absence of tGFP transcripts in all the tissues examined in ΔGPS animals (Figure 6).

## Discussion

In a multicellular organism, differential gene activity is the outcome of the initial decision that a cell makes whether a particular gene should be active or inactive followed by the modulation of the gene activity by the tissue/organ function. The data presented here demonstrates an overriding requirement for a GPS in the functioning of the *cryab* promoter both in cells in culture as well as in the transgenic mice (Figures 2, 3, 4, 5, and 6). Significantly, the GPS, unlike an enhancer, is position and orientation-specific (Figure 3).

The proximity of the GPS to the TSS and its orientation and position-specific function in the *cryab* promoter may suggest a simpler and direct mechanism of gene-specific control through its involvement with the development of transcriptional competence (or opening up of a promoter) [32]. The analysis presented here, however, does not preclude the existence of GPS-like elements at longer distances from the TSS in the eukaryotic promoters. It is interesting to note that sequences 100 bp upstream of the TSS have been previously suggested to control the lymphoid cell specificity of the expression from a κ-light chain immunoglobulin promoter [33].

The GPS identified here is either a binding site or a hub for transacting factor(s) or simply a landmark that dictates the physical state of the chromatin that allows gene activity [34-39]. The data presented in Figure 3 provides significant insight about the relationship between expression and the location of the GPS (distance of this sequence from the TSS). We know that changing the location of the GPS from the 5′ to the 3′ side of HSE-αB inhibits expression (Figure 3A, number 5). However, moving it more than 90 bp, 5′ upstream of the HSE (−64/−35), reduces the promoter activity marginally (Figure 3B, number 6). This tolerance to change in location, 5′ upstream of the HSE, becomes unacceptable when the GPS is moved more than 154 bp from the TSS, which results in drastic inhibition of the promoter activity (Figure 3B, numbers 2–5). A plot of the expression versus distance of the GPS from the TSS indicates a biphasic response, a slow less dramatic phase when at positions −71 and −154 and a fast declining component beyond −154 (Figure 3C). This data leads to two important inferences: (1) GPS must remain in proximity of the TSS, 5′ to the HSE to be functional, and (2) considering that 154 bp is roughly the size of DNA wrapped around a nucleosome bead, the GPS may have an influence on nucleosome spacing and/or the physical status of the nucleosomes in the vicinity of the TSS [35,36]. It is known that HSF4 (that binds to the HSE) has been reported to recruit BRG1 (Brahma-related gene 1), a member of the chromatin remodeling complex to *cryab* promoter [38,39] suggesting a possible function of the GPS via positioning of the nucleosomes in regulating access to the promoter.

While it remains to be established if trans-acting factor binding sites (including transcription factors) become functional only in the presence of a GPS, it is tempting to speculate that the apparent promiscuity in some DNA binding transcription factors, e.g., Pax6 [37,40] and possibly HSF4, may be brought about by gene-specific sequences like the GPS.

We have demonstrated recently that HSF4 is detected on the *cryab* promoter in ARPE cells indicative of its involvement in the expression from this promoter [18]. In light of this observation, the inhibition of the promoter activity upon deletion of GPS (Figure 3A, number 2) or upon change of its position (Figure 3A, number 5) suggests that HSF4 binding to HSE is not enough for eliciting gene activity but may also require a functional GPS. If this interpretation is extrapolated to the data obtained with transgenic mice, it is obvious that GPS may be essential for keeping the promoter open (active). This is borne out by the complete inhibition of *cryab* promoter activity in multiple tissues in transgenic mice made with constructs without the GPS (ΔGPS) in comparison with constructs that contained GPS (+GPS) (Figures 5 and 6). These data suggest that GPS may be obligatory for the activation of *cryab* transcription.

**Figure 7:**
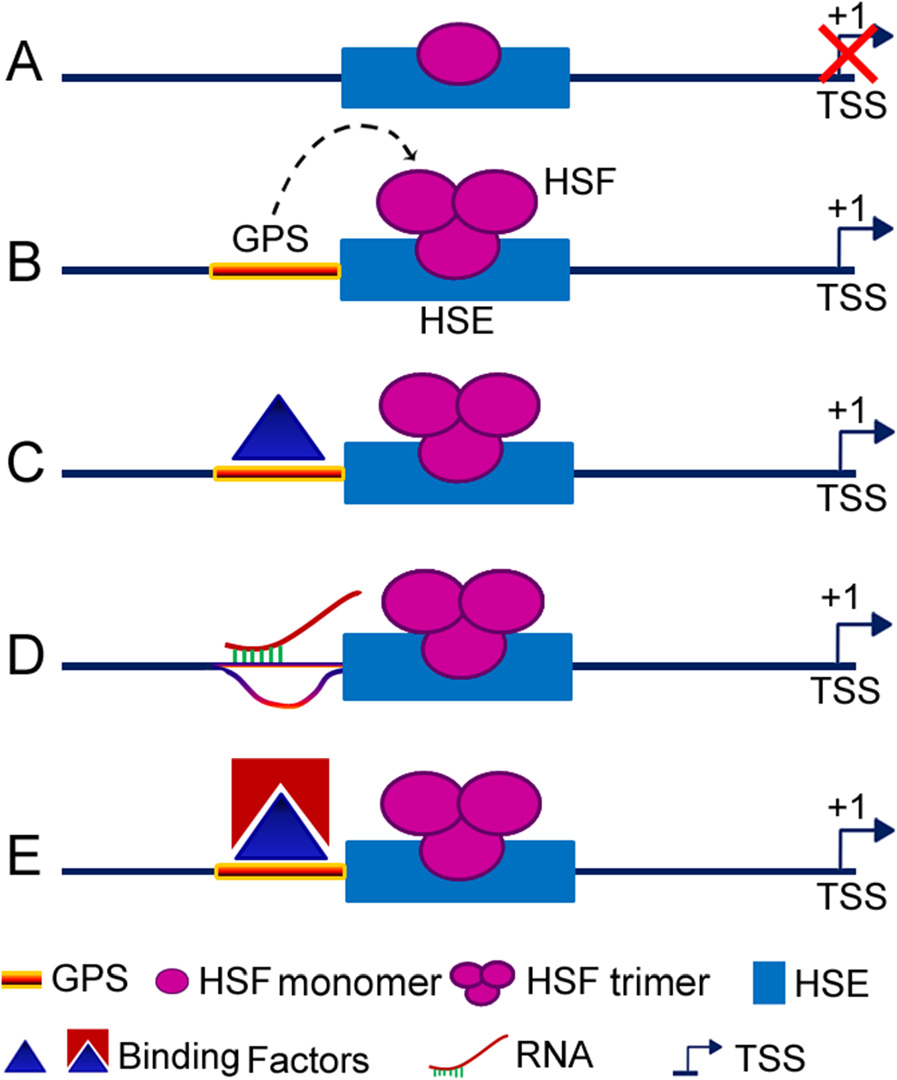
**Hypothetical schemes of GPS involvement in regulating *cryab *promoter activity.** The schematics shown are based on the observation that *cryab* promoter cannot function without the GPS. Without the GPS, the interaction of the HSF4 with the HSE is weak (indicated by monomeric interaction of HSF with HSE) and therefore non-productive (red X) **(*A*)**. GPS presence could simply enhance binding of HSF4 to the HSE by itself **(*B*)** or by binding to another transacting factor that could be a protein **(*C*)** or an RNA **(*D*)**. On the other hand, GPS could act as a chromosomal landmark for the open promoter. This may involve protein-DNA as well as protein-protein interactions **(*E*)** that would facilitate HSF4 binding to the HSE as well as opening of the promoter for transcriptional activity. We do not know what comes first: the involvement of the GPS, or the binding of HSF4 in the events that lead to the activation of the *cryab* promoter? The data presented in this investigation suggests that the involvement of the GPS must precede any event(s) that leads to *cryab* promoter activation.

**Figure 8:**
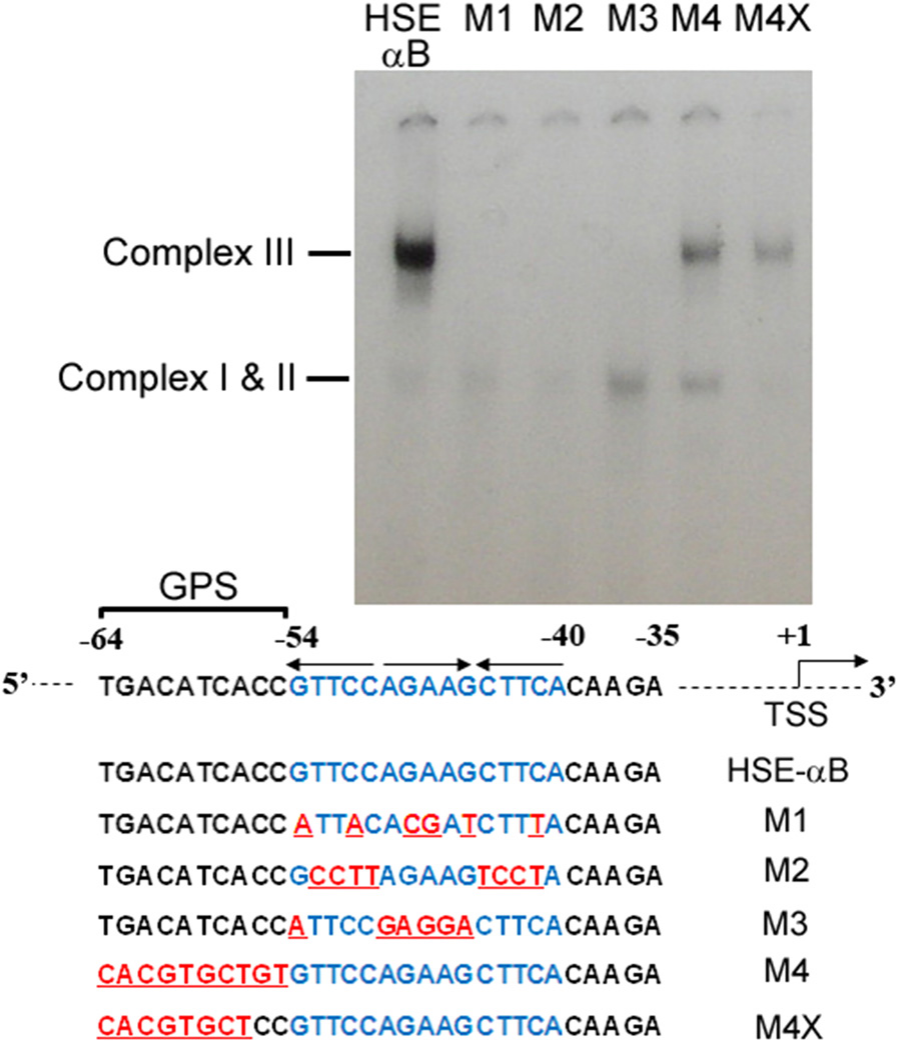
**Gel-shift analyses with **^**32**^**P-HSEαB and its mutants.** Post-natal day 10 rat lens nuclear extracts, which predominantly contain HSF4 [25], were used for these assays. Autoradiograph shows the complex III (*lane HSEαB*), the major complex, which contains trimeric HSF4, known to be associated with the active promoter. The two minor complexes (*I and II*), seen mostly when the heat shock promoter is not active, are not seen here clearly. *TSS* transcription start site. *M1–M3* are probes with mutations in one or two of the 5′-NGAAN-3′ motifs (*arrows*) of the trimeric HSE (*blue*), as expected [24,25] they do not bind HSF4. *M4* and *M4X* are two HSEαB mutant probes with alterations in the GPS. Both these probes have diminished HSF4 binding activity (complex III) when compared to wild-type probe, HSE-αB. All mutations are shown in *red* and are *underlined*. All the probes are 30-bp long and had comparable specific activities.

GPS is a gene-specific sequence. The mechanism of its involvement in regulating the heat shock promoter of the *cryab* gene can only be speculated at this time (Figure 7). It is possible that the efficiency of the binding of trans-acting factors to their cognate sites is dictated by the gene-specific promoter sequences, in which case it would explain how numerous binding sites all over the genome [4] would not be productive because of the absence of the GPS. That this may be the case is indicated by the early gel-shift studies, wherein we mutated the GPS in the ^32^P-HSE-αB probe and assayed its effect on the appearance of the HSE-HSF4 complex (complex III) in the nuclear extracts of the post-natal day 10 rat lens (Figure 8). Mutations introduced into the GPS significantly diminished the generation of the HSE-HSF4 complex formation (Figure 8). This *in vitro* data suggests that the GPS has a role in HSF4 binding. It is also possible that the gene-specific sequence binds a protein or an RNA that cooperatively impacts the productive binding of the transacting factor. Alternatively, the GPS may be modulated by local physiology and/or the developmental state via RNA or a protein binding factor. These speculations need to be investigated experimentally for a complete understanding of the role of gene-specific sequences in eukaryotic promoter regulation.

## Conclusions

We have identified a non-enhancer gene-specific, position- and orientation-dictated 10-bp sequence (GPS) within the heat shock promoter of the αB-crystallin gene that is required for expression from this promoter, in cultured cells as well as in transgenic mice. The data presented here brings up three important corollaries: (1) Since GPS is essential for expression even before transcription factors and/or enhancer sequences get involved, the initial activation of a gene may be dictated by the gene-specific information in the promoter DNA. (2) Because GPS sequences do not represent universal motifs, they cannot be computed. Thus, they may have to be identified through labor-intensive experimentation as done here on a gene-to-gene basis. (3) GPS sequences could become targets for manipulation of a cell's phenotype.

## Methods

### Construction of recombinant plasmids

A 940-bp DNA fragment, −896/+44 (upstream of the ATG in the first exon of the *αB* gene) was amplified from the rat (Sprague Dawley) genomic DNA using primers: F (forward) 5′-*ATAGTGCCGAGCCTCTTG*-3′ and R (reverse) 5′-*GGGAGTGGAAAGGAAAGAA*-3′ and cloned into pTOPO4 vector (Invitrogen, Carlsbad, CA, USA). This promoter sequence in pTOPO4 was used as the template for all downstream manipulations. The −896/+44 sequences represent complete rat *cryab* promoter. Beyond −896, there is another gene (*HspB2*), which is transcribed in the opposite orientation [41].

Two promoter constructs (αB-tGFP plasmids) were made: the truncated version (−64/+44) and the whole promoter (−896/+44) (Figures 2 and 3). These two constructs were made by amplifying two different lengths of the 5′-flanking region of the *cryab* promoter using one common downstream primer (+44R 5′-*ATCTAAGGATCCGATGGCTAGATGAGTGTAGAGTCG*-3′) and two upstream primers (−896 F 5′-*ATCTAAGAATTCACACCACCCAAAATAGTGCAGAGC*-3′ and *−*64 F 5′-*ATCTAAGAATTCTGACATCACCGTTCCAGAAGCTTC*-3′), respectively. These PCR products were gel purified and cloned into pTurbo-GFP-pRL (Axxora LLC., San Diego, CA, USA). The tGFP sequences start with an ATG. All mutations were introduced using commercially available site-directed mutagenesis PCR kit (Agilent, Santa Clara, CA, USA) and verified by sequencing. The sequences of the primers used for these manipulations are listed in Table 1.

**Table 1:** Oligonucleotides used in site- directed mutagenesis

**Primer set number**	**Sequence**	**Figure/construct number**
1	Sense: 5*'*-GCTCAGCCCAG*CGAAATTT*CAGCCTCTGCCCAGG-3*'*	Figure 2A/number 2
	Antisense: 5*'*-CCTGGGCAGAGGCTG*AAATTTCG*CTGGGCTGAGC-3*'*	
2	Sense: 5*'*-CCCTGACATCACC*ATTACACGATCTTT*ACAAGACTGCATATA-3*'*	Figure 2A/number 3
	Antisense: 5*'*-TATATGCAGTCTTGT*AAAGATCGTGTAAT*GGTGATGTCAGGG-3*'*	
3	Sense: 5*'*-ATAATAAAACCCC*CAGTGCTGTT*GTTCCAGAAGCTTCACAAGAC-3*'*	Figure 2A/number 5
	Antisense: 5*'*-GTCTTGTGAAGCTTCTGGAAC*AACAGCACTG*GGGGTTTTATTAT-3*'*	
4	Sense*:* 5*'*-CGAGCTCAAGCTTCGAATTC*CAGTGCTGTT*GTTCCAGAAGCTTCAC-3*'*	Figure 2A/number 9
	Antisense: 5*'*-GTGAAGCTTCTGGAAC*AACAGCACTG*GAATTCGAAGCTTGAGCTCG-3*'*	
5	Sense: 5*'*-CCGTTCCAGAAGCTTCA*CAAGC*CTGCATATATAAGGGGC-3*'*	Figure 2B/number 2
	Antisense: 5*'*-GCCCCTTATATATGCAG*GCTTG*TGAAGCTTCTGGAACGG-3*'*	
6	Sense: 5*'*-CCGTTCCAGAAGCTTCA*ACCGA*CTGCATATATAAGGGGCAGGC-3*'*	Figure 2B/number 3
	Antisense: 5*'*-GCCTGCCCCTTATATATGCAG*TCGGT*TGAAGCTTCTGGAACGG-3*'*	
7	Sense: 5*'*-CCTGACATCACCGTTCCAGAAGCTTCA*CTGCA*TATATAAGGGGCAGGCTG-3*'*	Figure 2B/number 4
	Antisense: 5*'*-CAGCCTGCCCCTTATATA*TGCAG*TGAAGCTTCTGGAACGGTGATGTCAGG-3*'*	
8	Sense: 5*'*-GCTGGGATAATAAAACCCC*GGTGATGTCA*GTTCCAGAAGCTTCACAAG-3*'*	Figure 3A/number 3
	Antisense: 5*'*-CTTGTGAAGCTTCTGGAAC*TGACATCACC*GGGGTTTTATTATCCCAGC-3*'*	
9	Sense: 5*'*-GCTGGGATAATAAAACCCC*CCACTACAGT*GTTCCAGAAGCTTCACAAG-3*'*	Figure 3A/number 4
	Antisense: 5′-CTTGTGAAGCTTCTGGAAC*ACTGTAGTGG*GGGGTTTTATTATCCCAGC-3*'*	
10	Sense: 5*'*-CCAGAAGCTTCACAAGA*TGACATCACC*CTGCATATATAAGGGGC-3*'*	Figure 3A/number 5
	Antisense: 5*'*-GCCCCTTATATATGCAG*GGTGATGTCA*TCTTGTGAAGCTTCTGG-3*'*	
11	Sense: 5*'*-GGGATAATAAAACCCCGTTCCAGAAGCTTCAC-3*'*	Figure 3B/number 2
	Antisense: 5*'*-GTGAAGCTTCTGGAACGGGGTTTTATTATCCC-3*'*	
12	Sense: 5*'*-GACACCTAGTTCTGACA*TGACATCACC*TATTGGTGGTCACAGCTCTCC-3*'*	Figure 3B/number 3
	Antisense: 5*'*-GGAGAGCTGTGACCACCAATA*GGTGATGTCA*TGTCAGAACTAGGTGTC-3*'*	
13	Sense: 3*'*-CCCTGGGGCTCAGCCCA*TGACATCACC*GGAAGATTCCAGCCTCTGCC-3*'*	Figure 3B/number 4
	Antisense: 5*'*-GGCAGAGGCTGGAATCTTCC*GGTGATGTCA*TGGGCTGAGCCCCAGGG-3*'*	
14	Sense: 5*'*-CTGGCTCCAGAGAACAAG*TGACATCACC*GATGGGGTGGGTGGGTGCC-3*'*	Figure 3B/number 5
	Antisense: 5*'*-GGCACCCACCCACCCCATC*GGTGATGTCA*CTTGTTCTCTGGAGCCAG-3*'*	
15	Sense: 5*'*-CTTTTCTTAGCTCAGTGAG*TGACATCACC*TACTGGGTATGTGTCACC-3*'*	Figure 3B/number 6
	Antisense: 5*'*-GGTGACACATACCCAGTA*GGTGATGTCA*CTCACTGAGCTAAGAAAAG-3*'*	
16	Sense: 5*'*-GGGGAGCTGGGATAATAA*TGACATCACC*AACCCCGTTCCAGAAGC-3*'*	Figure 3B/number 7
	Antisense: 5*'*-GCTTCTGGAACGGGGTT*GGTGATGTCA*TTATTATCCCAGCTCCCC-3*'*	
17	Sense: 5*'*-CCCCTGACATCACCCAAGACTGCATATATAAGGGG-3*'*	Figure 4/number 5
	Antisense: 5*'*-CCCCTTATATATGCAGTCTTGGGTGATGTCAGGGG-3*'*	
18	Sense: 5*'*-GCTGGGATAATAAAACCCCCAAGACTGCATATATAAGGGGC-3*'*	Figure 4/number 6
	Antisense: 5*'*-GCCCCTTATATATGCAGTCTTGGGGGTTTTATTATCCCAGC-3*'*	

The last column lists the associated figures. The mutated sequences are italicized.

### Cell culture and transfection experiments

ARPE-19 cells (ATCC, Manassas, VA, USA) [26] at 70% to 90% confluence were transfected with a mixture of experimental αB-tGFP plasmid DNA and pCMV-DsRed vector (Clontech, Mountain View, CA, USA) (50:1) using Lipofectamine2000 (Invitrogen). The pCMV-DsRed plasmid was used as an internal standard to normalize transfection efficiency. The experiments were done in triplicate and repeated three times.

### Transgenic mice and genotyping

The animal care and use protocol were followed as per institutional guidelines of the Animal Research Committee, University of California, Los Angeles, CA, USA. The whole promoter αB-tGFP constructs with or without GPS (construct with GPS shown in Figure 5A) were double digested with Xho I and Afl II to obtain a 2-kb fragment containing polyA signal (polyA is from the backbone of pTurbo-GFP-pRL plasmid). The fragment (αB-tGFP-polyA) was purified from the vector backbone and used for the generation of transgenic mice [42] at the UCLA Transgenic/Knockout Injection Facility. We generated five founders for + GPS and nine founders for ΔGPS constructs. Three lines each for + GPS and ΔGPS were examined for expression of the tGFP.

Genotyping was performed using PureLink™ Genomic DNA Mini Kit (Invitrogen) employing two primer sets (1F 5′-*GTGTCACCCTGCCAAATC*-3′, 1R 5′-*GCTCGAACTCCACGCCGTT*-3′; 2F 5′-*GCCACCATGGAGAGCGACGAGA*-3′, 2R 5′-*GATGCGGGTGTTGGTGTAG*-3′). To determine the copy number of αB-tGFP inserts in different transgenic strains, absolute qPCR assays were performed with 10-ng genomic DNA using the LightCycler 480 SYBR Master Mix (Roche, Indianapolis, IN, USA) [43,44]. The whole promoter αB-tGFP constructs were serial diluted as template, and four different amounts of DNA (1 ng, 100, 10, and 1 pg) were used in a 10-μl reaction for the generation of the standard curve. All reactions were done in triplicate. The qPCR thermal cycling conditions were as follows: 95°C for 5 min for hot start, followed by 45 cycles of 95°C for 15 s, 56°C for 20 s, and 72°C for 30 s. Specific primers were used in SYBR Green qPCRs were as follows: tGFP: 2F 5′-*GCCACCATGGAGAGCGACGAGA*-3′, 2R 5′-*GATGCGGGTGTTGGTGTAG*-3′. The average copy number in + GPS and ΔGPS transgenic mice were determined to be 6.2 and 18.0, respectively.

### Confocal microscopy and immunofluorescence

The whole embryos from + GPS (embryonic day 16, E16) and ΔGPS transgenic mice (embryonic day 15, E15) were fixed in 4% paraformaldehyde and processed as detailed previously [45] using anti-tGFP antibody (Axxora LLC., San Diego, CA, USA). Serial *z*-stack images were acquired from the whole eye, heart, and liver using a confocal microscope (FluoView 1000, Olympus, Tokyo, Japan) and processed using Adobe Photoshop Elements version 9.

### Immunoblotting and RT-qPCR

Mouse tissue extracts (post-natal, day 10 pups) were prepared in T-PER Protein Extraction Reagent (Pierce, Rockford, IL, USA). About 30 μg of protein/lane was electrophoresed on 4% to 12% SDS-PAGE gradient gels (Invitrogen) and transferred to nitrocellulose membranes for immunoblotting [26]. The reactive protein bands (anti-tGFP) were quantified using the LiCOR Odyssey dual wavelength IR system (LiCOR Biosciences, Lincoln, NE, USA). Gapdh was used as an internal control for all blots. Similar data was obtained with three lines of + GPS and ΔGPS transgenic lines.

Total RNAs were extracted 48 h after transfection of ARPE cells or from mouse tissues using TRIzol Plus RNA Purification System (Invitrogen, Carlsbad, CA, USA). RT-qPCR was conducted as described [18]. RT-qPCRs were performed in triplicate for each RNA sample in the Lightcycler 480 (Roche) (95°C for 5 min, followed by 45 cycles of 95°C for 15 s, 56°C for 20 s, and 72°C for 30 s). To calculate the relative change of tGFP expression, PCRs were normalized with reference to corresponding internal controls (DsRed RNA isolated for transiently transfected APRE-19 cells and Gapdh RNA for transgenic and wild-type mice tissues using the ΔΔCt method) and expressed as a percentage of the wild-type construct. Specific primers used were as follows: tGFP: F 5′-CTACCACTTCGGCACCTACC-3′, and R 5′-GATGCGGGTGTTGGTGTAG-3′; DsRed: F 5′-TACCTGGTGGAGTTCAAGTCC-3′ and R 5′-TCGTTGTGGGAGGTGATGT-3′. Gapdh: F 5′-GGTGAAGGTCGGTGTGAACG-3′ and R 5′-CTCGCTCCTGGAAGATGGTG-3′. We also assessed expression of tGFP in transfected ARPE cells (Figures 2, 3, and 4) with immunoblotting. This data mirrored the RT-qPCR data and is therefore not shown.

### Gel-shift

These experiments were done with ^32^P-labeled HSE-αB probes as previously described [24,25].

## Competing interests

The authors declare that they have no competing interests.

## Authors’ contributions

ZJ and RKG contributed equally to the experimental work. DCM helped with bioinformatics. SPB participated in the experimentation, supervised the project, and wrote the manuscript with ZJ and RKG. All authors read and approved the final manuscript.
